# MicroRNA-22 coordinates vascular and motor neuronal pathfinding via *sema4* during zebrafish development

**DOI:** 10.1098/rsob.210315

**Published:** 2022-04-06

**Authors:** Jiajing Sheng, Jie Gong, Yunwei Shi, Xin Wang, Dong Liu

**Affiliations:** School of Life Science, Nantong Laboratory of Development and Diseases; Second Affiliated Hospital; Key Laboratory of Neuroregeneration of Jiangsu and Ministry of Education, Co-innovation Center of Neuroregeneration, Nantong University, Nantong, People's Republic of China

**Keywords:** endothelial cells, guidance cues, microRNA, motor neuron, pathfinding

## Abstract

A precise guiding signal is crucial to orchestrate directional migration and patterning of the complex vascular network and neural system. So far, limited studies have reported the discovery and functions of microRNAs (miRNAs) in guiding vascular and neural pathfinding. Currently, we showed that the deficiency of miRNA-22a, an endothelial-enriched miRNA, caused dramatic pathfinding defects both in intersegmental vessels (ISVs) and primary motor neurons (PMNs) in zebrafish embryos. Furthermore, we found the specific inhibition of miR-22a in endothelial cells (ECs) resulted in patterning defects of both ISVs and PMNs. Neuronal block of miR-22a mainly led to axonal defects of PMN. Sema4c was identified as a potential target of miR-22a through transcriptomic analysis and *in silico* analysis. Additionally, a luciferase assay and EGFP sensor assay confirmed the binding of miR-22a with 3′-UTR of *sema4c*. In addition, downregulation of *sema4c* in the miR-22a morphants significantly neutralized the aberrant patterning of vascular and neural networks. Then we demonstrated that endothelial miR-22a regulates PMNs axonal navigation. Our study revealed that miR-22a acted as a dual regulatory cue coordinating vascular and neuronal patterning, and expanded the repertoire of regulatory molecules, which might be of use therapeutically to guide vessels and nerves in the relevant diseases.

## Graphical abstract

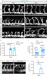

## 1. Introduction

During vertebrate embryogenesis, the formation of an architectural vasculature and nerve network is essential to ensure proper functioning. Blood vessels and nerves are exquisitely organized in nearly parallel patterns throughout the body. However, the mechanisms that regulate the endothelial cells (ECs) and neurons to follow specific migratory tracts during development have not been investigated sufficiently. Recent reports have demonstrated that vasculature and nerves possess common molecular factors to guide cell migration and pathfinding, and neuro-vascular communication is crucial for the development of both systems. Increasing evidence has demonstrated that several axon guidance signals including *Robos*, *UNC58*, *Plexins* and *Neuropilins* regulate vessel pattern and vice versa [1]. Apart from nerves, vessels also produce axon-guiding signals and vascular patterning signals [2,3]. In addition, proper neuronal axonal wiring in brain development also depends on the precise molecular regulation of neuro-vascular co-patterning [4]. Despite recent progress in the area of neuron control vascular guidance, the understanding of vessel contributions to nerves navigation and neuro-vascular communication is not yet fully explored.

MiRNAs are key post-transcriptional regulators of target mRNAs through binding at the 3′untranslated region (UTR). A miRNA often regulates a cluster of targets in a variety of biological processes. Of interest, more miRNAs have been reported to be widely distributed in nerves and vascular systems [5]. Thus, miRNAs are important candidate regulators of neuron and vessel development. A number of miRNAs have been reported to make roles during angiogenesis. However, the role of miRNAs in neuro-vascular development has not been extensively explored.

Semaphorins, categorized into eight classes, are a large family of transmembrane or secreted proteins. Semaphorins have been studied as axonal guiding cues for years and also found to participate in the processes of vascular development recently [6,7]. For example, as in the nervous system, autocrine endothelial Sema3A regulate EC migration, vascular navigation and patterning by binding to NRP1/Plexin [8]. Sema5A is a pro-angiogenic semaphorin that was suggested to regulate blood vessel remodelling and hierarchical organization during embryo development [9]. The class 4 subfamily of semaphorins have been found to be closely related to immunity and inflammation [10]. However, Sema4A, initially found as an activator of T cell-mediated immunity, was identified to promote EC apoptosis by inhibiting tyrosine phosphorylation of VEGFR2 [11]. In addition, Sema4D was found to increase EC migration through mDIA1/Src signalling pathway [12]. These results indicated that class 4 semaphorins may also participate in the behaviour of ECs. The Sema4c has been shown to play diverse roles in biological development, including neurogenesis [13], terminal myogenic differentiation [14] and epithelial mesenchymal transition (EMT) [15]. In addition, Sema4c and its receptor Plexin-B2 have been demonstrated to be expressed in the nervous system and ECs, but knowledge of their functions in vascular development is still limited [16].

Currently, our understanding of the association between axon guidance and vascular pattern is still superficial. Here, by taking advantages of the zebrafish as a model, we show that miR-22a derived from ECs plays a dual role in vascular and neuronal guiding by targeting *sema4c*. Knockdown of miR-22a caused zebrafish ectopic network in both blood vessels and nerves. The defect of the vascular patterning was mimicked by endothelial-specific reduction of the miR-22a. Interestingly, the phenotype in the nerve network was induced by both neuron-specific and EC-specific repression of miR-22a. We reveal that endothelial miR-22a regulates PMN axonal navigation via the exosome pathway. Furthermore, we identified *sema4c* as a direct target of miR-22a. These observations demonstrate that blood vessels mediate primary motor neuronal (PMN) pathfinding in zebrafish via exosome contained in miR-22.

## 2. Results

### 2.1. miR-22a is highly expressed in endothelial cells in the zebrafish

To investigate the expression of miR-22a in ECs, EGFP+ cells in *Tg(kdrl:EGFP)* zebrafish embryos at 22 hpf were isolated by fluorescence-activated cell sorting (FACS) as previously described [17,18]. Endothelial miRNA expression profiles were harvested by deep sequencing as previously reported [17,18]. The results of miRNAs sequencing were further verified using miRNA quantitative PCR, which showed that the expression level of miR-22a was comparable to that of miR-126, which is generally accepted as a highly expressed miRNA in ECs (electronic supplementary material, figure S1a,b). Furthermore, the expression profile of miR-22a in the zebrafish was analysed using whole mount *in situ* hybridization (ISH) with a digoxigenin-labelled probe. In the developing embryos, miR-22a hybridization signal was detected in the blood vessels, which was consistent with the TaqMan miRNA assay and sequencing result (electronic supplementary material, figure S1c).

### 2.2. Deficiency of miR-22a caused aberrant vascular networks

**Figure 1.**
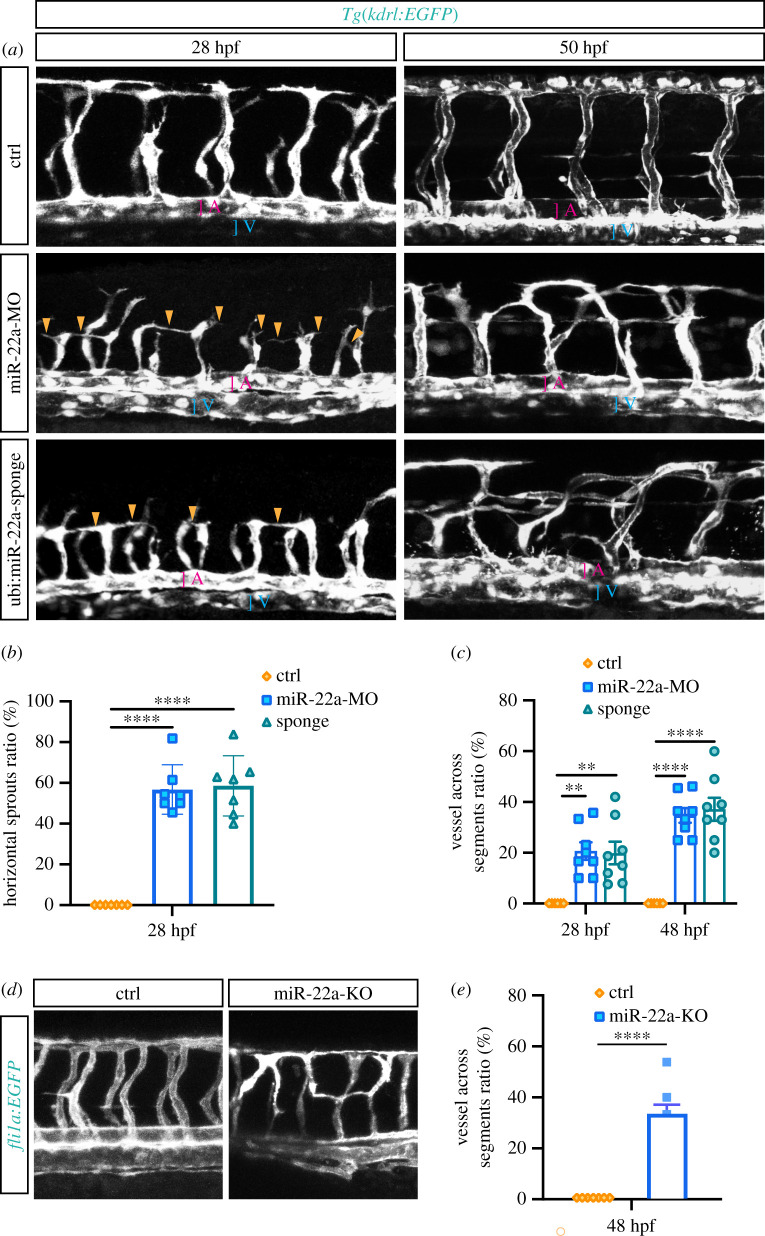
Deficiency of miR-22a caused aberrant vascular networks. (*a*) Confocal imaging analysis of control MO, miR-22a-MO and miR-22a-sponge-injected *Tg(kdrl:EGFP)* embryos at 30 hpf and 48 hpf. The arrowheads indicate the aberrant angiogenic sprouts. A: dorsal aorta; V: posterior cardinal vein. (*b*) Statistics of horizontal sprouts ratio in 30 hpf embryos injected with control MO (*n* = 7), miR-22-MO (*n* = 7) and miR-22-sponge (*n* = 7). One-way ANOVA, **** *p* < 0.0001. (*c*) Statistics of embryos with vessel across segments ratio in each group: control MO (*n* = 6), miR-22a-MO (*n* = 8) and miR-22-sponge (*n* = 8). One-way ANOVA, ** *p* < 0.01; **** *p* < 0.0001. (*d*) Confocal imaging analysis of control and miR-22a-KO *Tg(fli1a:EGFP-CAAX)* embryos at 48 hpf. (*e*) The statistics of embryos with vessels across segments ratio in each group: control (*n* = 7) and miR-22-KO (*n* = 7). *t*-test, ****, *p* < 0.0001.

Considering the high expression of miR-22a in zebrafish embryonic ECs, it is rational to speculate it might modulate the development of blood vessels. To investigate the function of miR-22a during blood vessel development, miR-22a morpholino antisense oligonucleotide (miR-22a-MO) was injected into single-cell-stage zebrafish embryos. This MO was designed to knockdown the expression of miR-22a through blocking Dicer and Drosha sites. The results of quantitative RT-PCR provided evidence that the injection of miR-22a-MO efficiently reduced the expression level of mature miR-22a (electronic supplementary material, figure S2). The morphology of ISVs at different stages of miR-22a-deficient zebrafish was examined by confocal microscopy. In contrast to a control group, at 28 hpf, the intersegmental vessels (ISVs) grew upwards halfway, then turned to horizontal sprouting (figure 1*a,b*). In 50 hpf, miR-22a-MO-injected embryos exhibited disorganized vascular networks (figure 1*a*,*c*). Specifically, the pattern of disorganized vasculature can be classified into three types. In the first case, the ISVs of miR-22a knockdown zebrafish are not confined to one somite, but connected to adjacent ISVs. In the second case, the ISVs grew upwards halfway, then reversely extended to dorsal aorta. In the third case, the ISVs grew halfway, then across somite to connect to opposite ISV or dorsal aorta. Furthermore, co-injection of miR-22a duplex significantly alleviated the disorganized ISV pattern, confirming the phenotype was a specific consequence of repression of miR-22a (electronic supplementary material, figure S3a,b). Moreover, we generated a transgene *Tg(ubi:miR-22a-sponge)*, in which the mature miR-22a was competitively buffered to compromise function, and the ISVs displayed a similar patterning defect to those of miR-22a morphants (figure 1*a*). To further confirm that miR-22a is required for the development of blood vessels, TALEN was used to knockout miR-22 in *Tg (fli1a:EGFP)* transgenic zebrafish line. Sequence analyses of the molecules amplified from the embryos microinjected with the group of T-1/T-2 and the group of T-3/T-4 mRNAs revealed that the mutated rate created by T-1/T2 was 46% (26 of 32), and that created by T-3/T4 was 23% (21 of 26), respectively (electronic supplementary material, figure S4). It was found that the disorganized vascular pattern was also observed in the ISVs of F0 knockouts, which was consistent with that in miR-22a knockdown zebrafish (figure 5*d*,*e*). Taken together, these results suggest that miR-22a is required for the well-ordered pattern of vascular networks.

### 2.3. Knockdown of miR-22a impaired the pathfinding of tip cells

**Figure 2.**
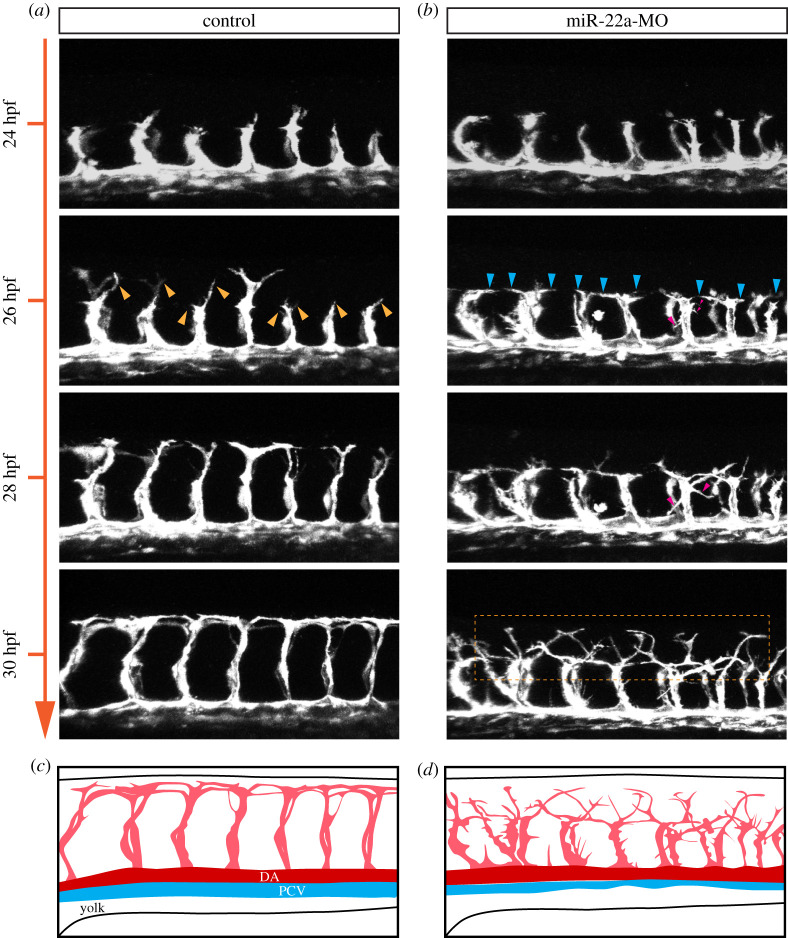
miR-22a regulates ISV tip cell behaviour. (*a*,*b*) Still images from *in vivo* time-lapse imaging analysis of *Tg(kdrl:EGFP)* embryos with HD detection setting. Time stages (hpf) are noted at the left side. The yellow arrows indicate the upward tip cells in the control groups. The blue arrows indicate the lateral tip cells. The red arrows indicate the downward tip cells. The rectangle box in dash line indicates the multidirectional filopodial extensions in the miR-22a deficiency embryos. (*c*,*d*) Diagrams of ISV morphology in control and miR-22a MO-injected embryos.

Endothelial tip cells have been proven to guide the proper wiring of nascent vessels through filopodia. To further confirm the role of miR-22a in governing endothelial tip cell behaviour, time-lapse imaging was performed in a *Tg(kdrl:EGFP)* zebrafish line. In the control, ISVs initiated to sprout from the dorsal aorta (DA) at 20 hpf, then grew upwards between somites to form dorsal lateral anastomotic vessel (DLAV) at around 30 hpf (figure 2*a*,*c*; Movie 1). In the miR-22a-deficient embryos, ISVs emerged from DA at around 22 hpf and arrived at the horizontal myoseptum. Although the process of ISVs sprouting was normal, the subsequent migration of tip cells displayed multi-directional filopodial extension and failed to reach the DLAV in time (figure 2*b*,*d*; electronic supplementary material, movie S2). Moreover, some tip cells stopped growing upwards, turned to the side, and even extended backwards (figure 2*a*,*c*). These results suggest that rather than being essential for ISV sprouting, miR-22 tends to regulate vascular patterning through affecting tip cell extension.

### 2.4. Deficiency of miR-22a caused aberrant axonal projection of PMNs

**Figure 3.**
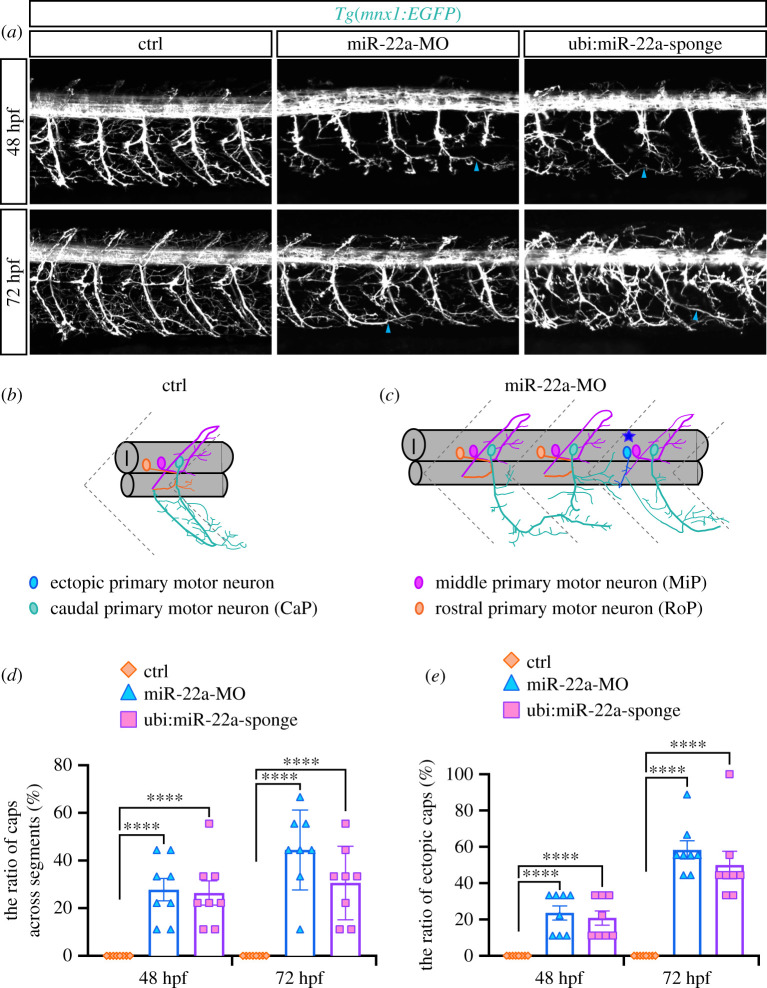
Deficiency of mir-22a caused aberrant axonal projection of PMNs. (*a*) Confocal imaging analysis of primary motor neurons in the control embryos, miR-22a morphants and *Tg(mnx1:GFP::ubi:miR-22a-sponge)* embryos at 48 and 72 hpf. Arrowheads indicate the CaPs across different segments. (*b*,*c*) Schematic diagram for primary motor neurons in the control embryos and miR-22a morphants. (*d*) Statistical analysis of the ratio of CaPs across different segments in the control (*n* = 8), miR-22a morphants (*n* = 8) and *Tg(mnx1:GFP::ubi:miR-22a-sponge)* embryos (*n* = 8) at 48 and 72 hpf; One-way ANOVA, ****p < 0.0001. (*e*) Statistical analysis of the ratio of aberrant axonal projection of CaPs in the control (*n* = 8), miR-22a morphants (*n* = 8) and *Tg(mnx1:GFP::ubi:miR-22a-sponge)* embryos (*n* = 8) at 48 and 72 hpf; one-way ANOVA, ****p < 0.0001.

Since miR-22a was shown to express in the neural system and is required for ISV pathfinding, we reasoned that it might play a role in axonal projection of neurons. In order to investigate whether miR-22a regulates neuronal pathfinding, we examined the morphology of PMNs in miR-22a knockdown *Tg(mnx1:GFP)*^ml2^ zebrafish embryos, whose PMNs were labelled with GFP using confocal microscopy imaging analysis. It was shown that absence of miR-22a caused dramatic developmental defects of PMNs (figure 3*a*). The axonal trajectories of caudal PMNs (CaPs) were significantly misled in the miR-22a morphants. Nearly one-third and half of CaPs axons were improper across neighbouring segments at 48 and 72 hpf respectively, with barely any in the control group (figure 3*a–d*). In addition, a unnormal motor neuron was found between the two CaPs in part spinal hemisegments of the miR-22a morphant, which also projected axons to the abdominal axial muscle (figure 3*c*). We defined this abnormal motor neuron as ectopic CaPs, whose number was quantified in different morphants at different stages (*n* = 8), and found the ratio of ectopic CaPs increased with the development of the zebrafish (figure 3*e*). In order to confirm the motor neuronal defects were specifically caused by miR-22a inactivation, we carried out confocal imaging analysis of *Tg(mnx1:GFP::ubi:miR-22a-sponge)*. The results revealed similar motor neuron phenotypes to those in the miR-22a morphants, including the altered CaP trajectory and ectopic PMN (figure 3*a*,*b*,*d*,*e*). Furthermore, we performed confocal time-lapse imaging analysis and found that the axonal trajectories of CaPs exhibited a tendency to cross the somite at around 36 hpf (electronic supplementary material, figure S5). From 48 hpf to 60 hpf, the axon of cap across the somite boundary continually extended parallel to the spinal cord (electronic supplementary material, figure S6).

### 2.5. miR-22a is required for both PMNs and ISV organization

**Figure 4.**
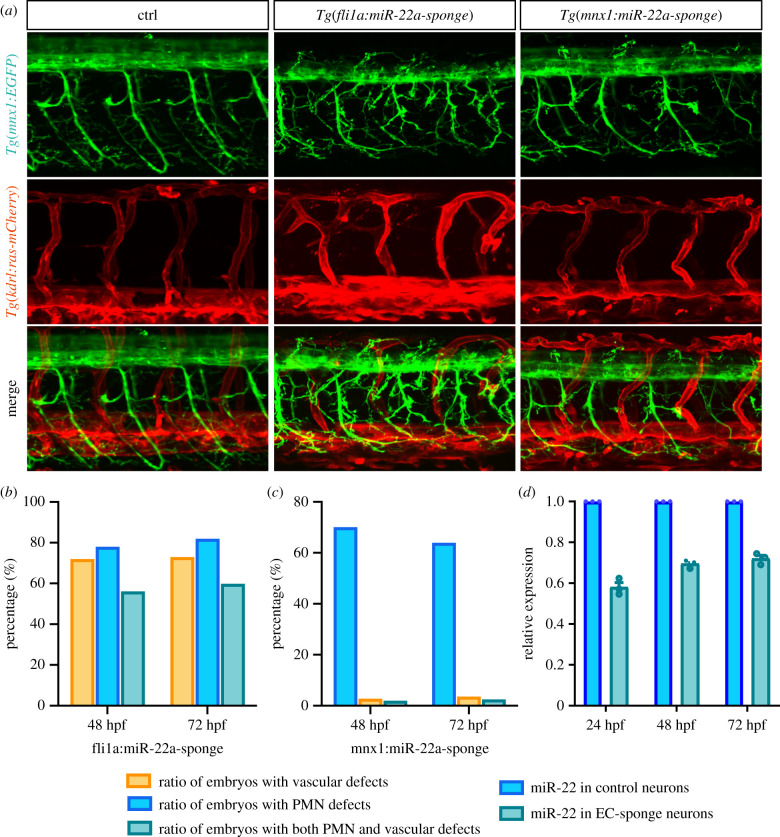
miR-22a regulates primary motor neuron and ISV pathfinding. (*a*) Confocal imaging analysis of fli1a:miR-22a-sponge-injected *Tg(mnx1:EGFP::kdrl:ras-mCherry)* embryos at 72 hpf. (*b*) Percentage of embryos with impaired ISV and PMN pathfinding phenotype in fli1a:miR-22a-sponge-injected embryos at 48 hpf and 72 hpf, respectively (*n* = 108; *n* = 120). (*c*) Confocal imaging analysis of huc:miR-22a-sponge-injected *Tg(mnx1:EGFP::kdrl:ras-mCherry)* embryos at 72 hpf. (*d*) Percentage of embryos with indicated phenotypes in huc:miR-22a-sponge-injected embryos at 48 hpf and 72 hpf, respectively (*n* = 112; *n* = 105). (*d*) Quantitative PCR analysis of miR-22a in control neuron cells and neuron cells sorted from embryo injected with ECs expressing miR-22a-sponge at 24 hpf (*n* = 3), 48 hpf (*n* = 3), and 72 hpf (*n* = 3). *t*-test; **p < 0.001.

To investigate the consequences of specific downregulation of miR-22a in ECs, we microinjected *Tg(fli1a:miR-22a-sponge)* constructs into single-cell-stage embryos, in which *miR-22a-sponge* was transiently expressed in ECs to block the function of miR-22a. The sponge contained seven repeats of the miR-22a antisense sequence, which inhibited miR-22a expression by chelating miR-22a. The expression patterns of fli1a:miR-22a-sponge were examined in *Tg(fli1a:EGFP)* zebrafish, which showed the specific expression of sponge sequences (electronic supplementary material, figure S7). Compared with the control group, embryos with ECs expressing *miR-22a-sponge* exhibited severe phenotypes of both PMNs and ISVs (figure 4*a*,*b*), as we found in miR-22a morphants. Furthermore, compared with the control groups, the expression of miR-22a was downregulated in neuron cells sorted from embryos with ECs expressing miR-22a-sponge (figure 4*d*). Thus, EC miR-22a is necessary for pathfinding of ECs and neurons in zebrafish. We then explored whether miR-22a in the neural system could regulate the pattern of nerves or vasculature as well. To address this point, we specifically reduced the level of miR-22a in neurons though microinjection of *Tg(mnx1:miR-22a-sponge)*, in which *miR-22a-sponge* expression was driven by the promoter of the neuron-specific gene. In contrast to specific downregulation of miR-22a in ECs, the function of miR-22a specifically in the neural system impaired PMN navigation, but less than 5% of those exhibited obvious vascular defects (figure 4*a*,*c*). Taken together, these results suggested that endothelial miR-22a were involved in both vascular and neuron pathfinding, while neuronal miR-22a only regulated neuronal navigation.

### 2.6. miR-22a targets *sema4c* in zebrafish embryos

**Figure 5.**
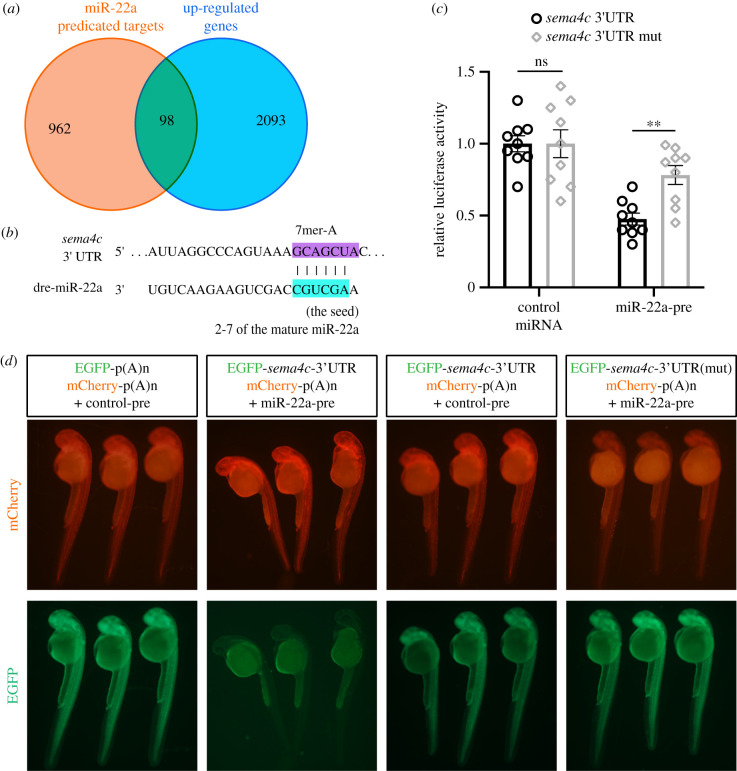
miR-22a directly targets *sema4c*. (*a*) Venn diagram of predicted target genes of miR-22a and transcriptomic upregulated genes. (*b*) *Sema4c* 3′-UTR target sites of miR-22a. (*c*) Overexpression of miR-22 (miR-22-pre) reduced *sema4c*-3′-UTR luciferase activity in HeLa cells (*n* = 9). Data are expressed as mean ± s.e. *t*-test; **p < 0.01. (*d*) EGFP sensors were co-injected with mCherry control as indicated. miR-22a-precursor injection reduced the EGFP levels in EGFP-*sema4c*-3′-UTR sensor (second column), whereas mCherry levels were unchanged. In the mutated sensor, no reduction in GFP was noted (experiments were repeated three times; for each group, around 10 embryos were analysed).

**Figure 6.**
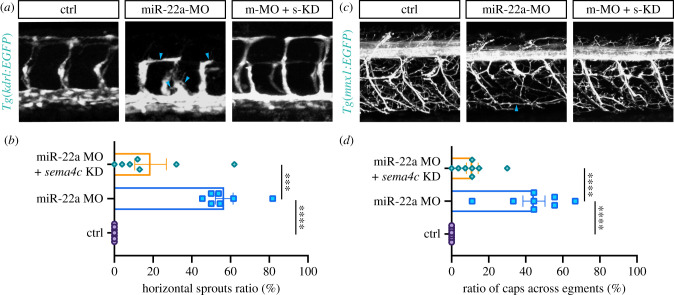
Reducing *sema4c* partially restored the defects of ISVs and PMNs in miR-22a-deficient embryos. (*a*) Confocal imaging analysis of blood vessels in control, miR-22a-MO, and miR-22a-MO + *sema4c* knockdown embryos at 30 hpf. (*b*) Statistics of horizontal sprouts ratio in control (*n* = 7), miR-22a-MO (*n* = 7) and miR-22a-MO + *sema4c* knockdown embryos (*n* = 7) at 30 hpf. One-way ANOVA; ***p < 0.001; ****p < 0.0001. (*c*) Confocal imaging analysis of control, miR-22a-MO and miR-22a-MO + *sema4c* knockdown *Tg(mnx1:EGFP)* embryos at 72 hpf. (*d*) The statistical analysis of the ratio of aberrant axonal projection of CaPs in the control (*n* = 8), miR-22a morphants (*n* = 8) and miR-22a-MO + *sema4c* knockdown embryos (*n* = 8) at 72 hpf; one-way ANOVA, ****p < 0.0001.

In order to identify the direct target of miR-22a, we carried out a series of experimental and *in silico* analyses. The RNA samples from control and miR-22a knockdown embryo at 30 hpf were used for transcriptomic sequencing, which revealed 2191 upregulated differentially expressed genes (DEGs) that might be affected by the repression of miR-22a. *In silico* analysis predicted that miR-22 potentially regulates thousands of genes in zebrafish using Targetscanfish (Release 6.2) and miRanda. A short list of overlapping target genes was selected from the transcriptomic DEGs and the predicted genes (figure 5*a*). These potential target genes were selected for possible involvement in the regulation of vascular development and axon guidance, including *kdr*, *sema4c*, *nppc*, *sema6d* and so on. Some class 4 semaphorins, such as *sema4a* and *sema4d* have been identified to participate in EC migration [11,12]. Recently, *sema4c* and its receptor Plexin-B2 have been demonstrated to express in nervous system and ECs, suggesting that *sema4c* may function as a regulatory cue during EC development [16]. *In silico* analysis predicted that the 3′-UTR of *sema4c* in zebrafish contained a potential miR-22a targeting site (figure 5*b*). To confirm the functional interaction of miR-22a and *sema4c*-3′-UTR, a luciferase assay and EGFP sensor assay in zebrafish was performed. The results indicated that the miR-22a precursor significantly inhibited the expression of *sema4c*-3′-UTR-EGFP but not *sema4c*-3′-UTR (mut), suggesting that miR-22a can directly target the zebrafish miR-22a-3′-UTR *in vivo* (figure 5*c*,*d*). By quantitative PCR analysis, the mRNA expression levels of sema4c in ECs and PMNs in miR-22a injection embryos were significantly increased (electronic supplementary material, figure S8). These results suggest that miR-22a targets the 3′-UTR of *sema4c* and thereby guides EC behaviour (figure 6).

### 2.7. Reducing *sema4c* partially restored the defects of ISVs and PMNs in miR-22a-deficient embryos

**Figure 7.**
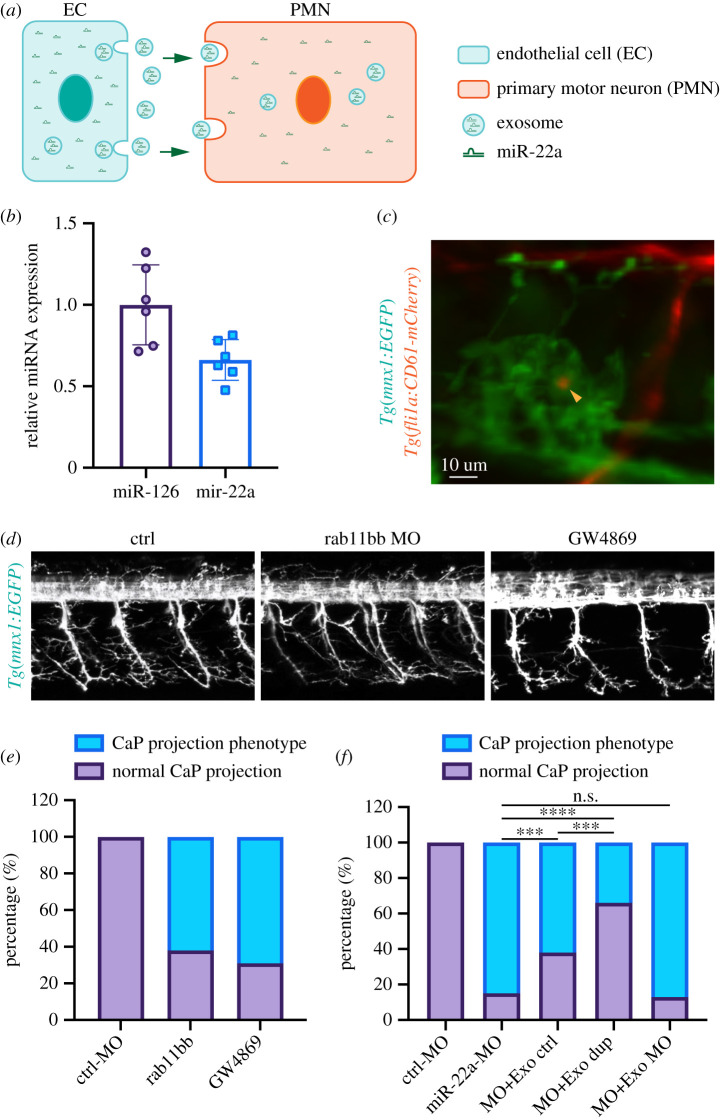
Endothelial miR-c22a regulates PMNs axonal navigation. (*a*) A possible working model for how blood vessels regulate primary motor neuronal pathfinding in zebrafish. (*b*) Q-PCR analysis of the miR-22 expression in the isolated exosome from HUVECs (*n* = 6). (*c*) Imaging analysis of *Tg(mnx1:EGFP::fli1a:CD61-mCherry);* arrowhead indicates the vesicle from endothelial cells. (*d*) Confocal imaging analysis of control, rab11bb MO injected and GW4869 treated *Tg(mnx1:EGFP)* embryos. (*e*) Percentage of embryos with indicated phenotypes in control (*n* = 30), rab11bb MO injected (*n* = 55) and GW4869 treated *Tg(mnx1:EGFP)* embryos (*n* = 38). (*f*) Percentage of embryos with indicated phenotypes in control (*n* = 18), miR-22 MO injected (*n* = 25), miR-22 MO co-injected with exosome isolated from HUVECs (*n* = 28), miR-22 MO co-injected with exosome isolated from HUVECs transfected with miR-22 duplex (*n* = 32) and miR-22 MO co-injected with exosome isolated from HUVECs transfected with miR-22 MO *Tg(mnx1:EGFP)* embryos (*n* = 23). Fisher's exact test, ****p < 0.0001; ***p < 0.001; ns, no significance.

Our results suggested that upregulation of *sema4c* was the likely cause of the defects of ISVs and PMNs in miR-22a-deficient embryos. If this was the case, the vascular and neuronal phenotypes of miR-22a-deficient embryos would be partially rescued by inhibiting the expression of *sema4c*. To investigate this possibility, *sema4c* was knocked down using a splicing-blocking morpholino (MO) in miR-22a morphants. The results of RT-PCR and sequencing provided evidence that the injection of sema4C-MO efficiently reduced sema4C expression (electronic supplementary material, figure S9). Then, the ISV and PMN phenotypes were examined using confocal imaging analysis. Knockdown of *sema4c* in this setting greatly normalized both ISV (figure 7*a*,*b*) and PMN projections (figure 7*c*,*d*). This result suggests that miR-22a regulates vascular and PMN pathfinding in zebrafish by directly targeting *sema4c*.

### 2.8. Endothelial miR-22a regulates PMNs axonal navigation

To explain why an endothelial-specific miR-22a knockdown embryo has defects in axons as well as blood vessels, and a neuron-specific miR-22a knockdown embryo also has axon pathfinding defects, we reasoned that the miR-22a of PMNs might be secreted by ECs. A possible working model was illustrated in a diagram (figure 7*a*). To verify this hypothesis, we carried out a series of experiments. Firstly, we found that miR-22a was highly expressed in exosome isolated from human umbilical vein ECs (HUVECs) by miRNA Taqman PCR analysis (figure 7*b*). Furthermore, vesicles labelled with *Tg(fli1a:CD61-mCherry)* were detected in the zebrafish developing PMNs (figure 7*c*), suggesting endothelial derived vesicle could be transported into PMNs. If the regulation of PMNs axonal navigation was involved in the exosome pathway, the blockage of the exosome formation would result in a phenotype of PMN axonal navigation. Numerous studies have shown that Rab11 is involved in the exocytic pathway, which is associated with post-Golgi membranes and secretory vesicles, and participates in exosome secretion by increasing intracellular calcium concentration [19–21]. In addition, GW4869, an inhibitor of exosome biogenesis/release, is the most widely used pharmacological agent for blocking exosome generation [22–25]. To test this hypothesis, we inhibited exosome generation through microinjection of the MOs targeting the blood-vessel-expressed Rab11(Rab11bb) and treatment of GW4869. Rab11-MO was designed to target the ribosomal initiation complex from the 5′ cap to block the translation of rab11bb. Its efficiency was confirmed by the constructs with MO binding site and reporter gene EGFP (electronic supplementary material, figure S10). It was found that both knockdown of Rab11 and GW4869 treatment resulted in axon pathfinding defects of PMNs that were similar to the phenotypes caused by endothelial miR-22a loss of function (figure 7*e*). In addition, we examined whether exosome isolated from ECs could rescue the axonal navigation phenotype of PMNs in miR-22a morphants. We found that co-injection of the exosome isolated from cultured HUVECs partially rescued the axonal navigation defects (figure 7*f*). Moreover, the percentage of phenotypes in embryos co-injected with exosome isolated from HUVECs, which were transfected with miR-22 duplex, was even less (figure 7*f*). We further revealed that co-injection of the exosome isolated from HUVECs, which were transfected with miR-22 MO, failed to normalize the percentage of axonal navigation defects (figure 7*f*). Taken together, these results suggested that endothelial miR-22a regulates PMN axonal navigation.

## 3. Discussion

In this study, detailed expression analysis showed that miR-22a was highly enriched in EC and neural system of zebrafish embryos. MO knockdown of miR-22a perturbed the pattern of ISVs and PMNs, indicating a requirement for miR-22a in directing endothelial and neuronal navigation during zebrafish embryonic development. Since the neuronal block of miR-22a specifically caused the axon pathfinding defects of PMNs, we excluded that the emergence of aberrant axonal navigation of PMNs was a consequence of vascular defects. Target prediction analysis indicated that miR-22a potentially regulates thousands of genes. With transcriptomic analysis, 2191 upregulated DEGs that might be affected by the repression of miR-22a were identified. A short list of overlapping target genes was selected from these DEGs and predicted genes. Combined with the sensor assay in zebrafish, *sema4c* was identified as a potential target. Then, the levels of *sema4c* were found to be elevated in miR-22a morphants by quantitive PCR analysis. In addition, inhibition of *sema4c* partially rescued the vascular and neuronal pattern in miR-22a morphants, suggesting that the observed phenotypes were caused by upregulation of *sema4c*.

The vascular system is so complicated that different cell types and signal factors are coordinated to regulate the developmental processes. In particularly, the precise wiring of the vascular network is regulated by several guidance cues that dynamically modulate endothelial cell-to-cell behaviour. Currently, accumulating evidence has indicated that guidance of blood vessel organization shares similar or common cues to those involved in nerves network, which share multiple parallels with the morphological and functional features of vascular systems. ECs can respond to multiple axon-guidance factors during vascular development. Conversely, vascular guidance signal may also regulate neuronal development [26]. For example, the work of Calvo *et al*. indicated that VEGFR3 signalling can influence neurogenesis as well as angiogenesis [27]. In addition, vessels can also produce axon-attracting cues for neuron development [28,29]. Sema4c is a member of class 4 semaphorins, which are a large family of secreted and membrane-bound cues that plays key roles in vascular patterning and axon guidance [30]. Sema4c has long been considered associating with neuronal migration, but recent report found that Sema4c and its receptor Plexin-B2 were also expressed in ECs [16]. In addition, Sema4c was reported to promote angiogenesis in breast cancer [31]. Although Sema4c is a transmembrane protein, its extracellular domain near the transmembrane portion can be cleaved [32]. Very few miRNAs have been reported to participate in blood vessel formation by targeting the components of semaphoring family [33]. Here we describe an endothelial-enriched miRNA, miR-22a, which plays a crucial role in zebrafish vascular and neuronal pattern via regulation of *sema4c*. Loss of function of miR-22a in zebrafish embryos led to aberrant pattern of ISV and PMNs, suggesting that miR-22a regulated ECs and neurons pathfinding. As the miR-22a expressed at a high level in the ECs, it is possible that the phenotypes we have identified were attributed to the miR-22a from ECs. We further investigated whether endothelial-derived miR-22a make dual roles in the vascular and neural systems. The consequences of specific downregulation of miR-22a in ECs and neuron cells were compared, respectively. It was found that all these phenotypes could be observed by inhibition of miR-22a in ECs. However, only aberrant neuronal pattern was phenocopied by loss of miR-22a in neuron cells. These data support the hypothesis that endothelial-derived miR-22a in zebrafish functions as regulatory signal regulates both vascular and neuronal branching and pathfinding. In addition, neuron-derived miR-22a could also regulate its patterning, while exerting no significant influence on the vascular network. We provide evidence to show that endothelial miR-22a regulates PMNs axonal navigation. These results give a possible explanation of why an endothelial-specific miR-22a knockdown embryo has defects in axons as well as blood vessels, and a neuron-specific miR-22a knockdown embryo also has axon pathfinding defects.

To date, limited studies have reported the discovery and function of miRNAs in vascular or neural patterning [34]. Accumulated reports have suggested that ECs and neurons can recruit various common guidance factors to control patterning of the complex vascular and neuronal networks. However, knowledge regarding the functional overlap between vascular and neuronal pathways, especially how the vessel contributes to neurovascular co-patterning, is still in its infancy. In the present study, we identified miR-22a, whose target gene *sema4c* is not only expressed in ECs, but also in neurons. Furthermore, miR-22a was revealed to play a crucial role in zebrafish blood vessel and neuronal patterning. Taken together, our findings provide a notable case that an endothelial-derived miRNA is attributed for regulating both ECs and neuronal pathfinding.

## 4. Material and methods

### 4.1. Zebrafish husbandry, breeding, anesthesia and euthanasia

The study was conducted conforming to the local institutional laws and the Chinese law for the protection of animals. All adult zebrafish (*Dario rerio*) were maintained under standard conditions in accordance with our previous protocols [35,36]. The *AB/WT*, *Tg(flk1:ras-mCherry)*, *Tg(kdrl:EGFP)*, *Tg(fli1a:EGFP)*, *Tg(fli1a:nEGFP)* and *Tg(mnx1:EGFP)* zebrafish used in this article have been described previously. Zebrafish embryos after 24 hpf were treated with 0.2 mM 1-phenyl-2-thio-urea to prevent pigment formation. Zebrafish embryos were anaesthetized with egg water/0.16 mg ml^−1^ tricaine (MS-222, Sigma) for live imaging. For euthanasia of zebrafish embryos, they were immersed in 300 mg l^−1^ tricaine for 10 min at 4°C.

### 4.2. Fluorescence-activated cell sorting

To compare the expression level of miR-22a in zebrafish neurons and ECs, *Tg(mnx1:EGFP)* and *Tg(Fli1a:EGFP)* larvae were used for neuron and EC sorting, respectively. To investigate the effect of miR-22a sponge manipulations on the expression level of miR-22a in ECs and neurons, *Tg(mnx1:EGFP)* and *Tg(kdrl:ras-mCherry)* larvae microinjected *mnx1:EGFP-miR22a-S* and *flia:EGFP-miR22a-S* were used for both EC and PMN sorting. Zebrafish embryos at 72 hpf were suspended by PBS containing 2% Fetal Bovine Serum (FBS) to remove the yolk. Then, embryos were digested using 0.25% trypsin at 28°C for 30 min followed by centrifugation. Collected cells were suspended with PBS (containing 2% FBS) and filtered with cell strainer (BD Falcon, 352340). The cells were used to sort ECs and neurons by a flow cytometer.

### 4.3. RNA isolation, reverse transcription, polymerase chain reaction, quantitative RT-PCR and RNA probe transcription

Total RNA of zebrafish embryos at various stages were extracted with TRizol according to the manufacturer's instruction (Invitrogen, Waltham, MA, USA) and genomic contaminations were removed by DNaseI. A quantity of isolated RNA was verified using gel electrophoresis and Nanodrop, followed by cDNA synthesis using Transcriptor First Strand cDNA Synthesis Kit (Roche) then was stored at −20°C. The custom-designed dre-miR-22a (accession: MIMAT0001788) TaqMan MicroRNA assays were purchased from Thermo Fisher Scientific Inc. (assay ID:004640_mat for dre-miR-22a, 000450 for hsa-miR-126, 002218 for hsa-miR-10b). The experiments were performed following the manufacturer's protocol. TaqMan MicroRNA Reverse Transcription Kit (4366596) was used for the microRNA reverse transcription. Quantitative RT-PCR was conducted in a total 20 µl reaction volume with 10 µl SYBR premix (TIANGEN). The relative RNA amounts were calculated with the comparative CT (2-DDCT) method and normalized with elongation factor 1-alpha (*ef1a*) as the reference. The primers for QRT-PCR are listed, for ef1a (accession: NM_131263.1):

 Forward primer: 5′-TGA TCT ACA AAT GCG GTG GA-3′;

 Reverse primer: 5′-CAA TGG TGA TAC CAC GCT CA-3′.

 Whole-mount *in situ* hybridization (WISH) with antisense RNA probes was synthesized as described previously [37]. The cDNA fragments used for miR-22a RNA probe transcription as template were amplified using the forward primer 5′-GAGGCCTCATCAGTTTGGAG-3′ and reverse primer 5′-TCTCACTGCTCTG CATGCTT-3′. Digoxigenin (DIG)-labelled sense and antisense probes were performed from the linearized pGEM-T-easy plasmids using the DIG RNA Labelling Kit (Roche).

### 4.4. Whole-mount *in situ* hybridization

Zebrafish embryos were harvested at various stages, fixed overnight in 4% paraformaldehyde (PFA), washed with PBST, dehydrated in methanol then stored at 4°C for subsequent use. The procedure for ISH follows our previous description [17]. For sectioning, above whole-mount embryos were transferred to Tissue-Tek OCT compound followed by being embedded in OCT blocks. The blocks were sectioned on a Leica RM2125 microtome at 10 µm.

### 4.5. Injection of morpholinos, microRNA precursor and construct

MO antisense oligomers were synthesized according to the manufacturer's protocol. The MO and miRNA precursor sequence is the following:

Dre-miR-22a-MO: 5′-AGCTTGCCAGTGAAGAACTGCTGCA-3′; Dre-miR-22a control MO: 5′-CACAGATTCGGTTCTACTGCCTTAA-3′; *sema4c*-MO:5′-CTTTTCTTGTCTGAACATACCTGTG-3′; rab11bb-MO: 5'-GCCATTTTAGACAAGCCGCCGCGTC-3′; miR-22a precursor :5′-GCUGACCUGCAGCAGUUCUUCACUGGCAAGCUUUAUGUCCUUGUGUACCAGCUAAAGCUGCCAGCUGAAGAACUGUUGUGGUUGGC-3′. MOs were prepared and injected into single cell stage embryos as described previously. The *Tg(fli1a:EGFP-miR-22a-sponge)* and *Tg(mnx1:EGFP-miR-22a-sponge)* construct was injected into one cell stage *Tg(kdrl:ras-mcherry)*-fertilized egg (1 ng per embryo).

### 4.6. Generation of miR-22a knockout zebrafish using TALENs

To make knockout zebrafish, four pairs of TALENs (T-1, T-2, T-3 and T-4) that target the pre-miR-22a sequence were designed using online tools (TAL Effector Nucleotide Targeter v. 2.0) as reported method [38,39]. The target sequence of T-1 was 5′- CCTGATCCCTGTTGTTTGGAGAAATATCAGGGACCGACTCCG TGAAGCC-3′; T-2 was 5′-GGCTGACCTGCAGCAGTTCTTCACTGGCAAGCT TTATGTCCTTGTGTACCAGCTA -3′; T-3 was CCTTGTGTACCAGCTAAAGCTGCCAGCTGAAGAACTGTTGTGGTTGGCTCTTTCCTGGC and that of T-4 was CAGCACACAAGATGGCAATACAGAGGTAACGACATTGTCAGCTCAACCCTTA (left and right arms are underlined). The expression plasmids of the TALENs were constructed using the ‘unit assembly’ method [40]. mRNAs were synthesized *in vitro* using the linearized PCS2-TALEN plasmids as templates with the SP6 RNA polymerase (Ambion), purified with the RNeasy MiniKit (Qiagen, Germany), and dissolved in ultrapure water treated with DEPC and stored in −80°C.

To test the activities of the TALENs, 1 nl of solution containing 250 ng µl^−1^ mRNA of above TALEN mRNA (talen-1-L/talen-1-R and talen-2-L/talen-2-R, talen-3-L/talen-3-R and talen-4-L/talen-4-R) was microinjected into zebrafish embryos at the 1–2-cell stage, respectively. The primers for amplifying the pre-miR-22a fragment containing the potential TALEN targeting site were F-miR-22a-1: 5′- TCCTTTCCCTCTCACTGCTC-3′ and R-miR-22a-1: 5′-ACCCAAATAAGGGCAA GAGG-3′. The PCR product was then cloned into pGEM-T easy vector (Promega) followed sequencing. Last, to generate the miR-22a knock-out zebrafish, the embryos were microinjected the mix of T-1, T-2, T-3 and T-4.

### 4.7. cDNA library preparation and sequencing

Total RNA was isolated and purified from 30 hpf zebrafish larvae using TRIzol Reagent (Invitrogen, USA). Then, RNA purity and integrity were verified by NanoDrop 2000 (Thermo Fisher, USA) and gel electrophoresis detection according to the protocol. A cDNA library was constructed using the TruSeq Ilumina RNA sample prep v2 kit by the manufacturer's protocol. The final cDNA library was sequenced on an Illumina HiSeq 4000 (Illumina, San Diego, CA, USA).

### 4.8. Identification of differentially expressed genes

Raw sequencing reads were assembled to the zebrafish reference transcriptome and genome (GRCz10 danRer10) using Bowtie2.0 and TopHat 2.0 (ref. 67). DEGs (control versus miR-22-MO) were identified according to the value of Z2 fold using DESeq tool.

### 4.9. Whole-embryo microRNA sensor assay in zebrafish

Whole-embryo microRNA sensor assay in zebrafish was carried out as described previously [17]. The coding sequences of EGFP and mCherry were cloned into the pCS2 + vector. The pCS2+-EGFP-*sema4c*-3′-UTR construct was generated by cloning 1121 bp 3′-UTR of the zebrafishmib1Mrna (ENSDARG00000079611) into the pCS2+EGFP vector, whereas pCS2+EGFP-*sema4c*-3′-UTR (MUT) was generated by inserting only nucleotides 611 bp of the *sema4c* mRNA, which lacks the fragments containing the miR-10 targeting sites. *sema4c* 3′-UTR and mut *sema4c* 3′-UTR were inserted between the *EcoRI*-*XhoI* restriction sites in the multiple cloning regions downstream of the EGFP gene. The following two pairs of primers were used for cloning the insertion fragment:

 *sema4c*-3′-UTR- EcoRI-left:

 5′ -CCGGAATTCTGTGGTAGTTGAGGTGCTATCT -3′;

 *sema4c*-3′-UTR-XhoI-right:

 5′- CCGCTCGAGACAGTGTGAGCCAGCCTTAA -3′;

 *sema4c* (mut)- 3′-UTR-EcoRI-left:

 5′- CCGGAATTCTTGTGGTAGTTGAGGTGCTATC -3′;

 *sema4c* (mut)- 3′-UTR-XhoI-right:

 5′-CCGCTCGAGACTGGGCCTAATACACTATTGT-3′.

The pCS2+-mCherry vector was used as a control. These three plasmids were linearized with Not1/Kpn1 and used as templates to synthesize the capped mRNAs using mMessage Machine (Ambion). The RNAs were injected into single cell stage embryos as described previously (35 pg per embryo).

### 4.10. EC culture, oligos transfection, exosome isolation and imaging

HUVECs culture and oligos transfection experiments were performed as described previously [17,41,42]. The exosome isolation was described in previous work [42]. For confocal imaging of nerves and blood vessel development in zebrafish, a specific stage of larvae was anaesthetized and embedded in 0.6% low melting agarose. Confocal images were acquired with a Nikon TI2-E-A1RHD25 confocal microscope or a Leica TCS-SP5 LSM. Images were processed using Imaris software. For the results of ISH, images were taken by an Olympus stereomicroscope MVX10.

### 4.11. Drug treatment

GW4869, exosome-secretase inhibitor, was purchased from Sigma-Aldrich (D1692) and dissolved in dimethyl sulfoxide (DMSO) at a final concentration of 50 µM. Egg water containing DMSO alone was used as control.

### 4.12. Statistical analysis

Statistical analysis was performed by student's *t*-test and one-way analysis of variance (ANOVA) using GraphPad Prism, in which *p*-values < 0.05 were considered statistically significant.

## Data Availability

All the high-throughput sequencing data generated in this study have been deposited in the GEO database. All the experimental materials generated in this study are available from the corresponding authors upon reasonable request.
